# Feasibility of delivering supervised exercise training following surgical resection and during adjuvant chemotherapy for pancreatic ductal adenocarcinoma (PRECISE): a case series

**DOI:** 10.1186/s13102-023-00722-3

**Published:** 2023-09-21

**Authors:** Malcolm Brown, Dominic O’Connor, Richard Turkington, Martin Eatock, Rebecca Vince, Claire Hulme, Roy Bowdery, Rebecca Robinson, Jonathan Wadsley, Anthony Maraveyas, Gillian Prue

**Affiliations:** 1 School of Nursing and Midwifery, Queen’s University Belfast Medical Biology Centre, 97 Lisburn Road, Belfast, BT9 7BL UK; 2 School of Health Sciences, The University of Nottingham, Nottingham, England, UK; 3 The Patrick G. Johnston Centre for Cancer Research, Queen’s University Belfast, Belfast, Northern Ireland UK; 4 The Northern Ireland Cancer Centre, Belfast Health and Social Care Trust, Belfast, Northern Ireland UK; 5 School of Sport, Exercise and Rehabilitation Sciences, University of Hull, Hull, England, UK; 6 Department of Health and Community Sciences, University of Exeter Medical School, Exeter, England, UK; 7 Pancreatic Cancer UK Research Involvement Network, London, England, UK; 8 Sheffield Teaching Hospitals NHS Foundation Trust, Sheffield, England, UK; 9 Department of Oncology and Metabolism, The Medical School, University of Sheffield, Sheffield, England, UK; 10 Hull York Medical School, University of Hull, Hull, England, UK

**Keywords:** Pancreatic ductal adenocarcinoma, Exercise, Feasibility, Functional fitness, Patient-reported outcomes

## Abstract

**Introduction:**

Pancreatic ductal adenocarcinoma (PDAC) is an aggressive neoplasm, with surgical resection and adjuvant chemotherapy the only curative treatment. Treatment-related toxicities place a considerable burden on patients although exercise training has shown promise is helping to manage such adversities and facilitate rehabilitation. The feasibility and safety of exercise training as a supportive therapy during adjuvant chemotherapy remains unknown.

**Methods:**

Patients with PDAC were screened post-surgical resection and enrolled in a 16-week, progressive, concurrent exercise programme alongside their chemotherapy regimen. Feasibility was the primary objective detailing recruitment, retention and adherence rates throughout as well as the safety and fidelity of the intervention. Secondarily, the impact on functional fitness and patient-reported outcomes was captured at baseline, post-intervention and 3-month follow up.

**Results:**

Eight patients consented to participate in this trial, with five proceeding to enrol in exercise training. Concurrent exercise training is feasible and safe during adjuvant chemotherapy and prevented an expected decline in functional fitness and patient-reported outcomes during this time.

**Discussion:**

This case series provides preliminary evidence that concurrent exercise training during adjuvant therapy is safe, feasible and well tolerated, preventing an expected decline in functional fitness, muscular strength and health-related quality of life (HRQoL). Given the adverse effects of treatment, these findings are promising and provide further evidence for the inclusion of exercise training as a standard of care for surgical rehabilitation and managing treatment-related toxicities. Future research should explore the impact of exercise training during neoadjuvant chemotherapy, with prehabilitation now standard practice for borderline resectable disease.

**Trial registration:**

ClinicalTrials.gov Identifier: NCT04305067, prospectively registered 12/03/2020, https://classic.clinicaltrials.gov/ct2/show/NCT04305067.

**Supplementary Information:**

The online version contains supplementary material available at 10.1186/s13102-023-00722-3.

## Introduction

Pancreatic cancer is an aggressive malignancy with poor survival outcomes. In 2020, 495,773 new cases of pancreatic cancer were reported globally, with 466,003 deaths [1]. Incidence and mortality rates have remained stable or slightly increased in many countries, to the extent that pancreatic cancer is projected to surpass breast cancer as the third leading cause of cancer death in Europe by 2025 [2]. Pancreatic ductal adenocarcinoma (PDAC) is the most commonly diagnosed neoplasm, accounting for more than 90% of cases [3]. Surgical resection remains the only curative treatment, with adjuvant chemotherapy administered as standard of care to improve survival rates. Although, these available treatment methods for PDAC are associated with chronic toxicities that impose a considerable physical and psychological burden [4]. Patients often experience debilitating side effects including reduced physical functioning, decreased skeletal muscle mass, heightened fatigue, gastrointestinal issues, pain and nausea [5]. Coupled with treatment-related toxicities, PDAC patients are at risk of developing associated comorbidities in sarcopenia and cachexia. In fact, cancer cachexia will affect up to 80% of pancreatic cancer patients during their disease course, with a significant proportion meeting cachexia criterion at diagnosis [6]. Even those eligible for resection can exhibit signs of cachexia, with reduced adipose tissue and muscle atrophy associated with poorer treatment responses to chemotherapy [6]. Ultimately, cancer cachexia impairs mobility and is strongly associated with morbidity and mortality [7]. Such toxicities and the risk of debilitating comorbidities, demands a need for adjunct therapies that counteract these complications.

Conventional exercise, particularly moderate to vigorous / high intensity aerobic and resistance training, delivered as part of rehabilitation or adjuvant therapy provokes numerous physical and psychological benefits that can alleviate several treatment-related toxicities and improve disease outcomes [4, 8–10]. Accumulating evidence suggests exercise training improves aerobic fitness, functional capacity, muscular strength and lean muscle mass [11–13]. The benefits of exercise training also extend to improving overall quality of life, pain, inflammation and cancer-related fatigue [14]. Thus, delivering exercise as a supportive therapy to adjuvant care could positively impact prognosis, given quality of life is an independent predictor of cancer survival and the associated treatment toxicities (e.g. fatigue) affects the vast majority of PDAC patients receiving chemotherapy [15, 16]. However, whilst the evidence favours exercise training as an important part of care, unlike other gastrointestinal cancers epidemiological evidence of the association between PDAC risk and / or progression and exercise remains limited, although some suggest greater volumes might decrease risk [17, 18]. The complexity of this disease, its treatment pathway and associated side effects / risk of comorbidities, provide a unique opportunity to test the effects of exercise training during treatment. Only recently have researchers diverted attention to these issues, but further work is now required to consolidate and enhance current understanding.

At present, clinical exercise trials in PDAC within the adjuvant setting are limited to a small selection of studies [19–23]. None of these trials included representation from the UK within their sample, so it is unclear how an exercise intervention might be implemented within the UK National Health Service. Concurrent exercise training has been shown to improve physical capacity, HRQoL, fatigue, sleep quality and importantly prevented muscular atrophy in a case sample [20]. Given body composition has been cited as a predictor of toxicity [24] and PDAC patients commonly suffer post-surgical weight loss and cachexia, this might prove clinically relevant. Recently, in a larger sample of 22 patients, supervised concurrent exercise training during adjuvant therapy proved safe and enhanced functional ability alongside muscular strength [23]. Clinically relevant individual changes were also noted for cancer-related fatigue and QoL, although body composition outcomes remained unchanged [23]. Such physiological improvements with exercise training could aid treatment tolerance, mitigate toxicities and arguably facilitate dose intensity, thus impacting the hard to shift endpoint of survival. Though speculative this downstream mechanism could arise from the direct biological effects of exercise on the tumour microenvironment [25] or from improved cardiovascular and metabolic functions, however the evidence base remains limited. In ESPAC4 trial, during adjuvant chemotherapy (Gem/Cap) only 54% of patients completed chemotherapy and a large proportion (47%) stopped treatment due to toxicity, with fatigue being the most commonly reported [26]. Exercise may help alleviate this and hence tolerability to treatment and therefore potentially survival. On the other hand, it could be argued that the fact that only 54% of patients completed chemotherapy highlights the need for a feasibility study in this disease. We propose that supervised, non-linear, concurrent training founded in the ‘principles of training’ could unlock the full therapeutic potential of exercise within this heterogenous population of PDAC patients. This approach involves manipulating intensity, duration and occasionally the frequency of training sessions to allow the training volume to continually progress across the entire programme. As there is considerable heterogeneity in this population, exercise programming should be equally individualised, to promote safety and optimise the efficacy of treatment for the individual. The aim of this trial is to establish the feasibility of delivering a prescribed, personalised, supervised exercise programme in PDAC patients undergoing adjuvant therapy, to improve outcomes and reduce symptom burden.

## Methods

### Participants

Participants diagnosed with PDAC, post-surgical resection and scheduled for adjuvant chemotherapy were screened for eligibility by clinicians within the Northern Ireland Cancer Centre, Belfast Health and Social Care Trust. Participants had no evidence of metastatic disease and no active prior malignancies (other than PDAC) within the last 3 years. Clinicians identified suitable participants and provided participant information packs at their chemotherapy planning clinic, with a view to enrolling in the exercise intervention after completing two cycles. The rationale for introducing exercise at this point, was to ensure participants tolerated chemotherapy well, prior to commencing exercise training. Participants were screened for recent and historical comorbid conditions that might contraindicate them from the exercise intervention (Table 1). Clinicians provided medical clearance to participate prior to chemotherapy cycle 3. Participants provided written informed consent to participate. At the time of exercise programming, all participants were treated with adjuvant gemcitabine / capecitabine or FOLFIRINOX (fluorouracil, irinotecan, leucovorin and oxaliplatin), bi-weekly for 12 cycles. The target sample size for this feasibility case series was 10 patients. Ethical approval for this trial was granted by the East of Scotland Research Ethics Committee (22-October-2019; Ref: 19/ES/0125). All the methods were conducted in accordance with relevant guidelines and regulations.

**Table 1:** Inclusion and exclusion criteria

Inclusion criteria
Histologically proven PDAC.
Complete macroscopic resection (R0 or R1 resection).
Currently receiving or planned to receive adjuvant chemotherapy (exercise to begin at cycle 3).
Prior malignancy active within the previous 3 years other than locally curable cancers that have been apparently cured, such as basal or squamous cell skin cancer, superficial bladder cancer, or carcinoma in situ of the prostate, cervix, or breast.
ECOG performance status 0–1.
Deemed medically fit by treating team to participate in exercise programme.
At least 18 years of age.
Medical clearance by treating clinician.
**Exclusion criteria**
Macroscopically remaining tumour (R2 resection or TNM stage IV disease).
Congestive heart failure or recent serious cardiovascular event.
Uncontrolled diabetes or another uncontrolled metabolic disease
Unstable angina
Chest pain while undertaking physical activity.
Any other active secondary malignancies
Other psychological, social or medical condition, physical examination finding or a laboratory abnormality that the Investigator considers would make the patient a poor trial candidate or could interfere with protocol compliance or the interpretation of trial results.

### Exercise intervention

The exercise intervention has been described previously [27]. Briefly, exercise training commenced following two cycles of adjuvant chemotherapy. Each participant received a personalised, supervised, progressive exercise programme for 16-weeks, running concurrently with chemotherapy. The programme comprised aerobic and resistance exercises, completed twice weekly under supervision by clinical exercise physiologists [MB and DO’C]. Participants were also encouraged to supplement supervised exercise with additional bouts of home-based aerobic exercise weekly. Prior to and following exercise, basic observations (i.e. blood pressure, oxygen saturation), self-rated fatigue and pain were obtained. This trial adhered to the principle of ‘autoregulation’, permitting reduced exercise when treatment-related side effects are heightened and supplemental exercise when they have subsided [28]. Upon entry participants completed 4-weeks of gradually progressive resistance exercise to familiarise then progressed to undulated exercise. The resistance exercise progressed in load from 12 to 6 repetitions, and 2 to 4 sets per exercise. Participants were encouraged to work beyond the prescribed exercise if treatment-related side effects were manageable. Typically, each supervised session commenced with a 10-min cardiovascular warm up, followed by 60 min of combined aerobic and resistance exercises. Aerobic exercise was performed on a cycling ergometer during supervised sessions, with brisk walking the preferred mode of exercise at home. Resistance exercise involved body weight, free weights and pin-loaded resistance machines to target the upper and lower extremities. Heart rate was monitored continuously throughout, using a Polar M200 watch, to ensure participants remained within the required heart rate zone (50–75% heart rate reserve). Onsite supervised resistance sessions were completed at a percentage of each participants 1-repetition maximum (1-RM) and separated by at least 48 h. Participants reported sessional rate of perceived exertion (RPE) using a 10-point scale. To minimise cross-interference between training modalities and to maintain variety, compliance and enjoyment, aerobic and resistance exercise timing alternated monthly. Each session was scheduled individually with reasons for cancellations or rescheduling noted, thus enabling intervention adherence to be calculated. Interruptions to the programme were documented if participants missed three consecutive sessions. To accommodate the recent COVID-19 pandemic, participants were offered a remotely supervised option using Zoom, but with obvious limitations in progression (e.g. dumbbells; resistance band exercises).

### Outcome measures

**Fig. 1:**
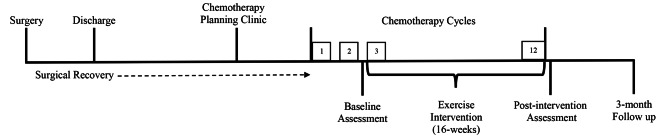
Trial timeline

Participants completed three outcome assessments at baseline (pre-chemotherapy cycle 3); post-intervention (chemotherapy completion) and at 3-months follow up. All assessments were performed by a clinical exercise physiologist. The trial timeline, from enrolment to completion, can be found in Fig. 1.

### Feasibility

Feasibility was determined by the number of participants recruited, retention and adherence rates. All variables were expressed as percentages, with adherence reflecting the number of sessions prescribed versus attended. Intervention fidelity (i.e. the prescribed dose and any deviations / escalations from the protocol) was determined and the rate of adverse events in response to exercise or treatment, from the point of informed consent. Adverse events were graded and coded according to the Common Terminology Criteria for Adverse Events (CTCAE).

### Anthropometric outcomes

Height and weight was determined using a free-standing stadiometer and calibrated laboratory scales respectively. Body mass index was derived from these measurements (kg/m^2^). Hip and waist circumference was measured in centimetres using a tape measure. Anthropometric assessments were captured by the same investigator throughout the trial.

### Physical fitness outcomes

Participants completed a timed six-minute walk test on a flat, indoor, 20-metre walkway. The six-minute walk test is a valid and reliable assessment in clinical populations and a surrogate measure of aerobic fitness [29]. Participants were instructed to walk briskly for the duration of the test. Heart rate response was monitored throughout, with perceived exertion rated at the end of the test. Muscular strength was assessed using a timed sit-to-stand test and 1-RM testing. For the timed sit-to-stand test, participants were instructed to rise from a seated position to standing upright and return to seating, without assistance, as many times as possible within 30 s. This 30-second sit-to-stand test is a valid and reliable measure of lower extremity strength [30]. 1-RM testing comprised a chest press, seated row and leg extension or leg press (not both). Prior to testing, participants completed a graded warm up, consisting of six and three repetitions at approximately 60% and 80% of their perceived maximum, respectively. For 1-RM testing, pin-loaded equipment was used and participants were instructed on correct breathing and lifting technique. 1-RM was determined within a maximum of five repetitions and sufficient recovery was provided between attempts. 1-RM is defined as the highest load that can be lifted, through the full range of motion, at one time.

### Patient-reported outcomes

HRQoL was assessed using a range of questionnaires that have shown to be valid and reliable in the cancer population [31]. The severity and impact of pain on daily living, over a recall period of 24 h, was measured using the Brief Pain Inventory Short Form [32]. HRQoL was measured using the EuroQOL 5-dimension 5-levels (EQ-5D-5L) and Functional Assessment of Cancer Therapy Hepatobiliary (FACT-Hep) questionnaires. The EQ-5D-5L questionnaire assesses HRQoL across five domains (mobility, self-care, usual activities, pain/discomfort and anxiety/depression) and provides a visual analogue scale for participants to self-assess their own health status [33]. The FACT-Hep is a 45-item HRQoL questionnaire assessing five domains (physical well-being, social/family well-being, emotional well-being, functional well-being; and additional concerns), with higher scores indicating improved quality of life [34]. Fatigue was assessed using the Functional Assessment of Chronic Illness Therapy - Fatigue (FACIT-fatigue) with higher scores indicating less fatigue [35]. Participants recalled and self-reported their physical activity levels (frequency and duration of vigorous intensity, moderate intensity, walking and sitting) during the previous 7 days, using the International Physical Activity Questionnaire (IPAQ) - Short Form [36].

### Data analysis

The number of participants screened, those accrued and those not willing to participate with reasons for ineligibility and non-participation were recorded. Participant attendance, compliance and completion rates for the intervention were analysed using descriptive analysis and reported as a percentage of their expected overall involvement. The acceptability of the measures of functional capacity and of the patient-reported questionnaires was reported using completion rates. Any observed changes in functional capacity and patient-reported outcomes from baseline were reported on an individual basis using descriptive statistics (i.e. mean).

## Results

### Eligibility and recruitment rate

**Fig. 2:**
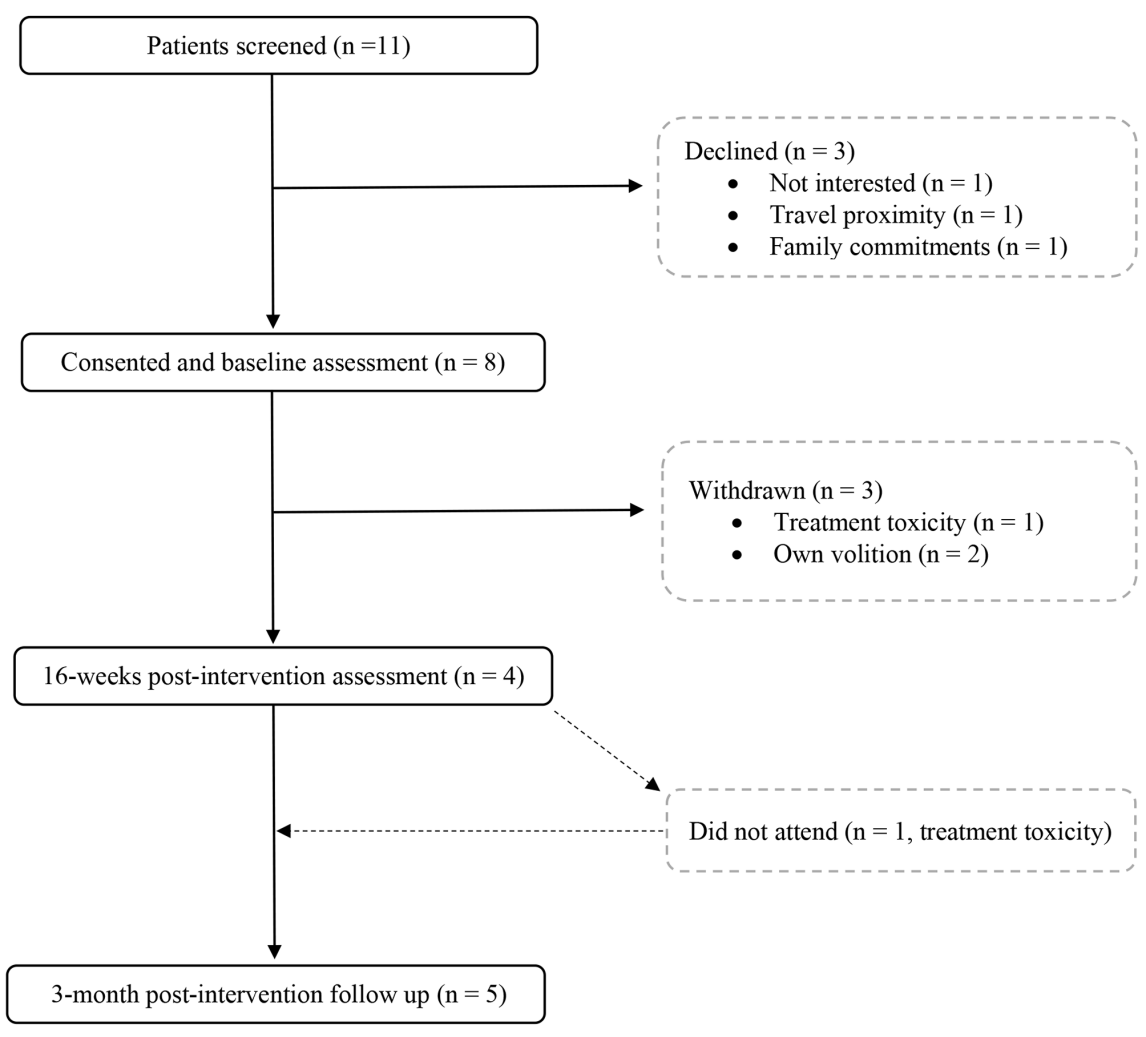
Patient flow diagram from screening to trial completion

In our 19-month recruitment window, from 3rd August 2020 to 31st December 2021, eleven participants with PDAC were screened, deemed eligible and approached by their treating clinician. Based upon regional statistics (1993–2020), the Belfast Health and Social Care Trust average 48 pancreatic cases per year (stages I-IV), with approximately 50% of these advanced cases and considerably fewer suitable for surgical resection (~ 20%) [37]. Positively, this suggests clinical gatekeepers approached the majority of those suitable for enrolment despite the challenging circumstances presented by the COVID-19 pandemic (e.g. restrictions on non-emergency surgery; suspended clinical trial recruitment). All eleven participants approached received a participant information pack and agreed to follow up. Eight participants (80% of original recruitment target) provided informed consent with five participants (63%) enrolling into the exercise intervention (Fig. 2, patient flow diagram). Three of the initial eight participants were withdrawn from the trial, in the time between consent and commencing the intervention (1 medically withdrawn; 2 withdrawn on their own volition citing personal reasons and proximity). The latter was offered a remote alternative, to enhance accessibility, but declined. Thus, the recruitment rate (the proportion enrolled versus eligible) for this trial was 46%. Three from the original eleven eligible participants declined the invite to participate citing differing reasons (not interested; travel proximity; family commitments). No demographic differences existed between those that agreed to participate and those that declined the invite to participate. The declining population was mixed in terms of gender (2 males, 1 female) and of similar age (68 ± 10 years). Therefore, the results presented are a case series of the five enrolled participants.

### Participant demographics

Participant demographics at baseline were recorded (Table 2). The age range for participants was 49–77 years. All patients were white, 40% were active smokers and 60% were retired. All five participants underwent surgical resection between June 2020 - August 2021 and were prescribed adjuvant chemotherapy between September 2020 - April 2022. The majority of cases increased or at least maintained their body mass (80%), with only participant 4 losing weight during adjuvant treatment. Measures of body composition can be found in Table 3.

**Table 2:** Patient characteristics at baseline

Variables	Mean (SD)
Age (years)	64 (11)
Height (cm)	171 (8)
Blood pressure (mmHg)	120 (15) / 75 (10)
Resting heart rate (bpm)	79 (7)
Oxygen saturation (%)	98 (2)
Respiratory rate (per min)	19 (2)
Comorbidities	
Diabetes	2
Tumour characteristics	
T1 T2 T3 T4 N0 N1 N2 N3 M0 M1	0 4 1 0 2 2 1 0 0 0
Days since surgery	125 (26)
Treatment	
Surgery Whipple procedure Distal pancreatectomy and splenectomy Laparoscopic	5 3 1 1
Chemotherapy	
Gemcitabine / capecitabine FOLFIRINOX Both (switch in treatment)	1 3 1
Race	
White	5
Current smoker	2
Employment status	
Professionally employed Retired	2 3

**Table 3:** Outcome measures

	Participant 1			Participant 2			Participant 3				Participant 4			Participant 5		
	Base	Post	3-M	Base	Post	3-M		Base	Post	3-M	Base	Post	3-M	Base	Post	3-M
Mass (kg)	69.2	75.2	79.7	58.6	DNA	64.3		79	82.1	83.5	79.5	74.7	76.6	71.4	72	72.4
BMI (kg/m^2^)	24.8	26.9	28.5	22.6	DNA	24.8		26	27.1	27.5	23.6	22.2	22.7	24.4	24.4	24.7
Hip (cm)	91	104	100	97	DNA	99		97	102	104.5	99	95.5	95	98	95	95
Waist (cm)	85.5	97	97	82	DNA	86		93	99	103	95	94.5	93	89.5	88	86
6-min walk (m)	270	300	320	140	DNA	160		506	549	615	447	523	480	500	560	590
Timed sit to stand (reps)	10	13	12	8	DNA	8		15	15	14	13	13	13	12	16	20
Chest press 1RM (kg)	38	45	52	Om	DNA	Om		61	70	72	Om	Om	Om	52	58	61
Seated row 1RM (kg)	43	65	75	Om	DNA	Om		61	82	93	Om	Om	Om	65	80	79
Leg press 1RM (kg)	Om	Om	Om	Om	DNA	Om		96	123	160	Om	Om	Om	Om	Om	Om
Leg extension 1RM (kg)	45.5	65	79	Om	DNA	Om		Om	Om	Om	Om	Om	Om	107	131	116
FACT-G Physical Social Emotional Functional FACT-Hep	94 27 26 24 17 159	101 25 28 24 24 167	85 22 25 22 16 141	34 2 20 6 6 60	DNA DNA DNA DNA DNA DNA	48 8 28 7 5 90		86 25 24 21 16 152	73 23 20 20 10 136	101 25 26 22 28 169	108 28 28 24 28 175	100 22 28 24 26 162	98 22 24 24 28 164	102 23 28 23 28 169	104 26 28 22 28 175	107 27 28 24 28 179
FACIT-Fatigue	42	47	32	11	DNA	5		47	36	48	48	20	30	45	49	52
BPI																
Severity Interference	0 0	2.75 0.57	1.5 1	4 6.86	DNA DNA	4.25 8		1.75 0.57	0 0	0 0	0 0	0 0.3	2.5 0	0 0	0 0	0 0
EQ-5D-5 L																
Health state VAS	11,222 90	11,122 75	33,332 70	22,433 50	DNA DNA	43,444 25		11,111 70	11,111 80	11,111 95	11,111 85	21,111 90	11,121 90	11,111 90	11,111 90	11,111 100
IPAQ																
Vigorous (d•wk^− 1^) Time (min•day^− 1^) Moderate (d•wk^− 1^) Time (min•day^− 1^) Walking (d•wk^− 1^) Time (min•day^− 1^) Sitting (h•day^− 1^)	0 - 0 - 7 Unsure 3	3 60 2 120 7 60 6	0 - 0 - 4 30 8	0 - 0 - 1 10 7	DNA DNA DNA DNA DNA DNA DNA	0 - 0 - 0 - 12		0 - 0 - 5 60 8	2 90 0 - 2 30 9	0 - 0 - 5 60 5	0 - 7 120 2 30 6	1 20 5 20 4 30 5	0 - 1 20 4 30 6	1 120 1 180 2 20 4	2 45 4 60 7 60 4	2 60 1 360 2 60 8

Abbreviations: Base, baseline; BMI, body mass index; BPI, brief pain inventory; d•wk^− 1^, days per week; DNA, did not attend; EQ-5D-5L, EuroQol 5 dimensions 5 levels; FACT-G, functional assessment of cancer therapy - general; FACT-Hep, functional assessment of cancer therapy – hepatobiliary; IPAQ, International Physical Activity Questionnaire; post, post-intervention; min•day^− 1^, mins per day; Om, omitted; 3-M, 3-month follow up; 1RM, 1-repetition maximum. 1RM testing: Participants completed 1 out of 2 leg exercises based on preference, not both. Participant 2 1RM testing omitted due to frailty; participant 4 1RM omitted due to out of hours attendance for outcomes.

### Retention and adherence rates

Five participants (63%) proceeded to the intervention and follow up at 3 months. Intervention delivery commenced on the 7th December 2020 and ceased on 29th April 2022. Participant 1 completed the trial in August 2021, attending 28 out of 32 supervised sessions during his adjuvant chemotherapy (88% adherence). Participant 2 completed the trial in September 2021 (baseline assessment and follow up outcomes only). This participant became non-contactable after baseline and despite persistent efforts to re-establish contact and seek an alternative method of delivery, participant 2 did not complete any supervised exercise sessions during adjuvant chemotherapy. Participant 2 cited a series of treatment-related toxicities for this absence, consistent with a change in chemotherapy regimen (switched from FOLFIRINOX to gemcitabine / capecitabine after cycle 2). Participant 3 completed the trial in November 2021, attending 28 / 32 supervised sessions (88% adherence). Participant 4 was the next to complete the trial in July 2022. Due to distance from the facility this participant availed of the hybrid option, predominantly completing remotely supervised exercise via Zoom (n = 17) and in person supervision (n = 5) prior to each chemotherapy cycle. Participant 4 completed 22 / 32 supervised sessions (69% adherence). The final participant completed the trial in July 2022, attending 16 / 32 supervised sessions (50% adherence). Overall, 80% (4 / 5 participants) were able to complete the exercise programme.

### Intervention fidelity

Exercise training was interrupted 5 times during the entirety of delivering the intervention, predominantly due to treatment-related toxicities (e.g. low cell counts). In total, participants missed 34 sessions (27%) and the intended programme was modified on 49 occasions (38%) (Table 4). Positively, the exercise dose was escalated on 53 occasions (41%), allowing participants to recover some of the altered dose. In terms of the aerobic component participants were prescribed a cumulative dose of 1080 min and completed 686 ± 362 min (available in the supplementary material). One participant exceeded the planned dose during the 16-week intervention (participants 3: 1142 min), while three participants completed less than the prescribed dose (participants 1, 4 and 5: 410, 380 and 810 min respectively). Regarding resistance training, three participants opted to attend regular supervised sessions at the treatment site and were prescribed a cumulative dose of 150,580 ± 33,936 kg, completing 131,782 ± 42,270 kg (available in the supplementary material). All three participants progressed to completing undulated resistance training and coped well with the requirements, lifting more than 100,000 kg during the 16-week intervention (participant 1–103,177 kg; participant 3–180,336 kg; participant 5–111,834 kg). The sessional breakdown of lifted versus prescribed is available in the supplementary materials.

**Table 4:** Tolerability to exercise training

Variable	*N*	Pct. (%)
Exercise interruption	5	-
Health-related		
Treatment-related Non-health related	4 1	- -
Missed sessions Health-related Conflicting appointments Cell counts / infection Fatigue Nausea Diarrhoea Non health-related Work commitments Holidays / vacation Technology issues Too busy Other / no reason	34 19 2 5 3 3 6 15 3 4 3 2 3	27 15 2 4 2 2 5 12 2 3 2 2 2
Dose modification Health-related Fatigue Muscle pain Non health-related Time constraints Other / no reason	49 21 18 3 28 1 27	38 16 14 2 22 1 21
Dose escalation	53	41

### Adverse events

No intervention-related adverse events occurred during the trial, however a number of treatment-related adverse events were recorded, resulting in missed exercise training (Table 4). Common treatment-related side effects included fatigue, low cell counts, nausea and diarrhoea. Exercise training was permitted with fatigue, but carefully managed and encouragingly all four participants were still able to exercise. However, exercise programming was paused with more severe side effects until they subsided.

### Physical outcomes

At baseline, all five participants completed a 6-min walking test and a 30-second sit-to-stand test. Participant 2 did not complete post-intervention outcomes, but the remaining four participants all completed the same outcomes following the intervention. At 3-month follow up, all participants completed the same physical tests. Aside from participant 2, who stopped walking prior to the expiration of the 6-min duration, these physical tests were well tolerated. The mean walking distance at baseline, post-intervention and 3-month follow up was 431 ± 110, 483 ± 123 and 501 ± 134 m respectively (participant 2 outcomes omitted due to incomplete attendance). All four participants that engaged with the intervention improved their aerobic fitness at post-intervention and at 3-month follow up. In terms of the timed sit-to-stand test, all actively engaged participants improved or at least maintained their lower extremity muscular strength at post-intervention and 3-months. Participants 1, 3 and 5 also completed 1RM testing at baseline, post-intervention and 3-months. All three tolerated this testing well and improved their upper and lower extremity muscular strength at post-intervention and again at 3-months (individual outcomes in Table 3).

### Patient-reported outcomes

The patient-reported outcomes were acceptable and feasible. All participants understood and completed the questionnaires fully (no missing data), suggesting these measures are suitable. The outcomes vary on an individual basis immediately post-intervention and at 3-months, with some improving and some declining (individual outcomes in Table 3). Participants 1 and 5 reported a meaningful improvement in fatigue post-intervention, while participants 3 and 4 reported heightened fatigue at the same time point compared to baseline. Positively, fatigue levels subsided for participant 3 at follow up. HRQoL (i.e. FACT-G and EQ-5D-5 L scores) followed a similar trend and are equally variable overall, although some positive findings are observed individually for health state and self-rated health outcome. For example, participant 1 reported an improved overall FACT-G score post-intervention (as a result of a meaningful improvement within the functional domain), while participant 3 reduced their overall FACT-G, due to decreased scoring across all 4 domains. Encouragingly, participant 3 reported much improved quality of life at 3-months, outscoring in all 4 domains. Conversely, on the self-rated EQ visual analogue scale, participant 1 reported reduced health at post-intervention and 3-months, while participant 3 reported an improvement at post-intervention and again at 3-months.

## Discussion

In terms of feasibility, this trial demonstrates that patients with PDAC are receptive to considering exercise training while undergoing adjuvant treatment and the approach by their treating clinician is an important factor in this process. Of those approached, 73% (8 out of 11 patients) provided informed consent, with three withdrawing prior to commencing exercise. Patient dropout is commonplace in clinical trials examining PDAC patients, especially those adopting exercise training. A recent systematic review estimates retention rates of 71–90% for pancreatic cancer in the neoadjuvant setting [4]. The retention rate in this current trial was short of this estimate at 63%, although the small sample and recruitment climate (e.g. COVID-19) are important considerations. Positively and in agreement, a recent trial in patients with localised or metastatic pancreatic cancer undergoing non-surgical treatment, reported a post-intervention retention rate of 50% [23]. Home-based exercise may prove a more accessible alternative, with higher retention rates reported in pancreatic cancer [19], however most often greater improvements are seen under direct supervision [38, 39]. Intervention adherence was high in four of the five participants enrolled, with attendance ranging from 50 to 88%. Consistently, adjuvant trials in PDAC have reported similar levels of adherence [20, 21, 40]. Three participants that lived in the vicinity of the exercise facility regularly attended twice-weekly sessions, while one participant residing further afield adopted a hybrid model of supervised and home-based exercise training using Zoom teleconferencing, highlighting the utility of this model during adjuvant therapy for PDAC. Supervised exercise training was scheduled individually, depending on participant availability, at a hospital-based exercise facility. The exercise training facility was co-located at the site of adjuvant therapy, providing continuity in treatment and perhaps resulting in high adherence. Pragmatism and flexibility in scheduling was necessary to maintain adherence, given the significant and persistent adverse effects experienced during chemotherapy.

While participants endured numerous treatment-related side effects throughout, an important finding of this case series is no intervention-related adverse events were recorded. This suggests this exercise programming is safe as an adjuvant therapy to support patients during treatment and agrees with previous findings [19, 20, 40]. Taken alongside retention and adherence data, it appears this exercise programme is feasible and could be delivered on a larger scale. However, implementation as a standard of care for PDAC patients and the most efficient methods of delivery will require greater attention. The supervision of exercise training by a suitable qualified professional, whether in person or remotely monitored, was fundamental to ensure safety, retention and compliance, but practically may be difficult to implement given economic and personnel constraints on the UK National Health Service. This stresses the need for cost effectiveness studies to demonstrate the cost benefit of employing exercise specialists, within this setting.

Tolerability to exercise training during adjuvant therapy is a vital consideration for PDAC patients. This trial demonstrates the fidelity of delivering aerobic and resistance exercise training, during adjuvant therapy. Obviously, throughout the course of treatment it is to be expected that patients face adversities and as a result the prescription is modified or sessions are missed, however these patients remained engaged and returned to exercise training once these subsided. It is reassuring that participants also had the capacity to escalate sessions and complete more than required on a given day, thus recovering some of the missed dose. This is a consistent finding, previously reported in localised and advanced prostate cancer [41, 42] and highlights the necessity of ‘autoregulation’ to allow patients that are willing and able, to recover missed exercise. During methodological design, we anticipated this might be the case and accounted for treatment toxicities within the undulating nature of the programme, however it appears that as toxicities accumulated the aerobic component proved more difficult to achieve (Fig. 2, supplementary materials, weeks 7–16). Although this should be interpreted with caution, as it was not universally noted, but worth emphasising for future, larger trials in this population.

Four of the five participants in this case series improved or at least maintained their physical fitness and muscular strength. Positively these adaptations persisted at 3-month follow up, suggesting exercise training prompted a chronic adaptation and / or instilled a motivation to continue exercising beyond the cessation of the trial. Patients often experience reduced cardiorespiratory fitness and negative changes in body mass during treatment, associated with a greater risk of post-surgical morbidity and mortality [43, 44]. The fact that exercise training prevented this functional decline and the subsequent negative consequences is promising and appears to be a consistent finding, even though the quality of the evidence base is limited [40]. A case study reported six months of supervised, concurrent exercise training during adjuvant therapy improved physical capacity, muscular strength and importantly prevented muscular atrophy [20]. Further confirmation of this beneficial adaptation was reported during a six-month adjuvant trial of resistance training, comparing supervised to home-based settings. Wiskemann and colleagues [21] reported greater improvements in strength with supervised exercise, seemingly making it more effective. However, adherence was greater with home-based resistance exercise, presenting a unique debate as to which is better (i.e. participation or the gain itself). A preoperative trial in PDAC has recently shown home-based exercise training is indeed effective in preserving skeletal muscle health compared to usual care controls [45]. This highlights the potential utility of a hybrid model of allowing patients to partake in both options and equally avail of the benefits of exercise training. Given PDAC patients suffer debilitating treatment-related side effects that persist into survivorship, these physical improvements could attenuate such toxicities and assist in enhancing HRQoL.

There is growing recognition that HRQoL measured through patient-reported outcomes (PROs) are crucial variables in oncology trials [46]. PROs provide a holistic perspective of the wider impact of treatment from the patients personal experience. This enriches understanding of subjective symptoms difficult to accurately measure and informs future care, especially during surgical rehabilitation and while enduring adjuvant therapy. The PROs measured are inconclusive, partly due to the sample size and the ‘snapshot’ nature of the outcomes (time point on a given day where symptoms might be exacerbated or reduced). Some participants reporting improved fatigue, health state and HRQoL, while others reported no change or negative scores. Given HRQoL normally declines, as a result of numerous treatment-related toxicities, improved or unchanged outcomes should be viewed in a positive light, while negative responses are to be expected over the course of treatment. Encouragingly, several other trials have reported improved HRQoL and decreased fatigue following both supervised and home-based exercise as well as at follow up [19, 20, 22, 45]. While the results are variable overall and largely a result of treatment [47], exercise training should still be prescribed on an individual basis to cater for those within this patient population that might respond and continue to avail of these benefits, while preventing the rate of decline frequently observed during treatment.

As body composition is associated with the risk of cachexia and thus morbidity and mortality in PDAC, it is reassuring the majority of participants involved increased or at least maintained their body mass. Similarly, resistance exercise training in the same population increased overall body weight by 3.2% [21]. Only participant 4 (home-based exercise programme) lost weight during adjuvant treatment, but his BMI remained healthy throughout. A recent trial demonstrated negative outcomes in terms of body composition at 3-months post-surgery [48], so the fact body composition improved and prevented functional decline is positive. A limitation of our study was that we did not measure the differentials of body composition, meaning we cannot propose with any certainty this exercise programme might increase lean mass, however this has been reported previously alongside comparable improvements in muscular strength [20]. While we are acutely aware, one might not inform the other in this present case series, cautionary optimism remains especially as the maintenance of skeletal muscle during chemotherapy is a reliable prognostic factor in survival [49].

Finally, all four participants regularly participating in the exercise programme subjectively reported increased levels of moderate to vigorous physical activity post-intervention. At a time when patients often face heightened treatment-related side effects, this change is favourable and potentially assisted in managing such toxicities. This is a consistent finding within the literature [50, 51] suggesting that exercise training during treatment can actively encourage behavioural change and enable patients to adopt habitual physical activity, even in the short term. However, upon the removal of supervised exercise whereby participants self-managed their own programme, physical activity levels decreased at 3-month follow up. Even with this reduction, physical outcomes improved possibly highlighting a training effect or instead the limitations of subjectively reported physical activity levels [52]. Regardless, this stresses the importance of instilling an exercise or health care professional to lead programming beyond the cessation of adjuvant treatment or instead refining the referral pathway to community-based programmes, so exercise provision and support can continue to be provided.

### Limitations and future directions

This trial was originally planned as a UK-wide, multi-centre intervention, but due to the impact of the COVID-19 pandemic and funding limitations, only one site was successfully opened during the recruitment timeframe. Thus, the single centre limits our ability to generalise the findings UK-wide, but indeed illustrates potential trends with exercise training during treatment for PDAC and highlights a need for scaled up research. A sufficiently powered sample would permit greater analysis that could determine if any changes were statistically significant and clinically meaningful. A potential strategy to increase accessibility, would be to accommodate home-based, remotely supervised exercise as a delivery choice to determine its effectiveness on survival, recurrence and treatment-related toxicities. While this case series did facilitate this option through a hybrid model, it was in response to the COVID-19 global pandemic, which in itself created a unique limitation whereby reduced surgical capacity and reduced in person contact made recruitment more difficult. Encouragingly this trial continued to recruit, illustrating the desire of people with pancreatic cancer to participate in an exercise programme. In keeping with the methodological design of future trials, the ‘principles of training’ should form the foundation of exercise programming as well as incorporating a comparator control / usual care arm, to assess whether an exercise intervention has any meaningful impact beyond the current standard of care. A definitive RCT, determining the efficacy of exercise on PROs and survival, is the logical next step. On a related note, it would appear that resistance exercise may be more tolerable in this population and should receive increased attention in future investigations. As toxicities persist long into survivorship, outcome measures should be reflective of this and longitudinally followed up to determine the chronic impact of exercise training in this population as well as its impact on the risk of recurrence. Further, forthcoming studies should attempt to assess the differentials of body composition, particularly lean mass, given the risk of sarcopenia and cachexia in this population. Positively, this trial was feasible and effective for the participants involved but requires a suitably qualified and experienced exercise or health care professional to deliver and individualise the prescribed dose and oversee the immediate transition of patients into survivorship, which makes its implementation into routine NHS practice challenging. Finally, future research is also encouraged to explore the mechanisms of action of exercise training in improving disease trajectory and survival for PDAC, particularly its influence on tumour growth and anti-tumour immunity. A recent pioneering trial reported aerobic exercise restricts PDAC tumour growth in mice, mediated by IL-15 signalling (a reputed ‘myokine’) and upregulation of anti-tumour immunity, ultimately sensitising tumours to therapy [25]. It is conceivable that the tumour microenvironment evolves under the influence of factors in systemic circulation and cellular crosstalk following exercise, given intracellular perturbations in skeletal muscle can stimulate the secretion of numerous factors that exert autocrine, paracrine and endocrine effects on tumours. This theoretical hypothesis provides the foundation for translational research in humans.

## Conclusion

This current case series provides preliminary evidence that concurrent exercise training during adjuvant therapy for PDAC patients is safe, feasible and well tolerated and may prevent expected declines in functionality, muscular strength and HRQoL during chemotherapy. Given the effects of surgical resection and cumulative effect of adjuvant chemotherapy on outcomes, a larger definitive trial of exercise training in this model is necessary, perhaps alongside the inclusion of a home-based or hybrid alternative. Nonetheless, this trial provides an insight and good starting point in the design of future studies. Including exercise training as a standard of care for surgical rehabilitation and during adjuvant therapy could significantly reduce morbidity and mortality in PDAC and better equip patients to endure further treatment if necessary.

### Electronic supplementary material

Below is the link to the electronic supplementary material.

Supplementary Material 1

## Data Availability

Data generated during this study is available from the corresponding author on reasonable request.
