# Supplementary material for: Feasibility of delivering supervised exercise training following surgical resection and during adjuvant chemotherapy for pancreatic ductal adenocarcinoma (PRECISE): a case series
Source: BMC Sports Sci Med Rehabil. 2023 Sep 21;15:116. doi: 10.1186/s13102-023-00722-3 (PMC10514993; doi:10.1186/s13102-023-00722-3)
Supplement: Supplementary file 1 — Supplementary Material 1 [file 13102_2023_722_MOESM1_ESM.docx]

**Fig. 1** Total cumulative dose of planned and completed aerobic exercise training.

**Fig. 2** Aerobic exercise training volume per week (prescribed versus completed).

**Fig. 3** Total cumulative dose of planned and completed resistance exercise training.

**Fig. 4** Resistance exercise training volume per session (planned versus completed).
